# Green Synthesis of CHA Zeolite from Expanded Perlite Waste for Rapid and Selective Pb^2+^ and Cd^2+^ Removal

**DOI:** 10.3390/molecules31091377

**Published:** 2026-04-22

**Authors:** Changchang Fan, Binyu Wang, Pan Xu, Jiaojiao Lv, Haoyang Zhang, Zixuan Liang, Wenfu Yan

**Affiliations:** 1 State Key Laboratory of Inorganic Synthesis and Preparative Chemistry, College of Chemistry, Jilin University, Changchun 130012, China; fancc24@mails.jlu.edu.cn (C.F.); xupan24@mails.jlu.edu.cn (P.X.); lvjj20@mails.jlu.edu.cn (J.L.); haoyang22@mails.jlu.edu.cn (H.Z.); liangzx25@mails.jlu.edu.cn (Z.L.); 2 Changchun Institute of Optics, Fine Mechanics and Physics, Chinese Academy of Sciences, Changchun 130033, China; wangbinyu@ciomp.ac.cn; 3 State Key Laboratory of Advanced Manufacturing for Optical Systems, Changchun 130033, China; 4 University of Chinese Academy of Sciences, Beijing 100049, China

**Keywords:** expanded perlite waste, **CHA** zeolite, Pb^2+^ removal, Cd^2+^ removal

## Abstract

The increasing release of non-biodegradable heavy metals, particularly lead (Pb^2+^) and cadmium (Cd^2+^), poses severe risks to ecosystems and human health. Herein, we present a sustainable “treating-waste-with-waste” strategy that simultaneously addresses heavy-metal contamination in water and the accumulation of expanded perlite waste. Expanded perlite waste was directly converted into a high-purity, low-silica **CHA** zeolite via a simple, one-pot, template-free hydrothermal conversion. The resulting sodium-exchanged material (Na-CHA-p) demonstrated excellent Pb^2+^ and Cd^2+^ removal performance, featuring ultrafast adsorption kinetics (reaching equilibrium within 5 min for both ions), high adsorption capacities (555.6 mg·g^−1^ for Pb^2+^ and 211.0 mg·g^−1^ for Cd^2+^), and superior selectivity. This study demonstrates an efficient pathway for the high-value utilization of perlite waste and highlights the strong potential of waste-derived **CHA** zeolites as advanced adsorbents for heavy-metal wastewater remediation.

## 1. Introduction

Rapid industrialization has led to increasing emissions of heavy metals into the environment, among which Pb^2+^ and Cd^2+^ are of particular concern due to their widespread occurrence and high toxicity. These contaminants are released primarily via industrial effluents, mining activities, and other anthropogenic processes [1,2]. Pb^2+^ is non-biodegradable and readily accumulates in the human body; even low-level exposure can cause neurological damage, renal dysfunction, and other serious health effects. Similarly, Cd^2+^ exposure is associated with multi-organ toxicity and an elevated risk of carcinogenesis [3]. The effective removal of Pb^2+^ and Cd^2+^ from wastewater is therefore of critical importance.

A variety of technologies have been developed for Pb^2+^ and Cd^2+^ removal, including chemical precipitation, membrane separation, electrochemical treatment, and ion exchange [4,5,6,7,8]. Nevertheless, chemical precipitation and membrane-based processes often suffer from high energy consumption, complex operation, or secondary pollution, while electrochemical methods can be costly and difficult to scale. In contrast, ion exchange is widely regarded as a highly efficient and environmentally benign approach, offering strong selectivity and operational simplicity for targeted heavy-metal removal [9,10].

Zeolites are crystalline microporous aluminosilicates that are extensively applied in ion exchange, gas separation, and catalysis [11,12,13]. Their excellent performance arises from a negatively charged framework and a well-defined pore architecture, which together enable efficient cation exchange. To date, more than 260 distinct zeolite framework types have been recognized by the International Zeolite Association Structure Committee (IZA-SC), each designated by a unique three-letter code, such as **CHA**. Among them, low-silica zeolites are particularly attractive for heavy-metal adsorption because their high aluminum content provides a high density of exchangeable sites [14,15].

**CHA**-type zeolites, composed of double-6-ring (d6r) units and small eight-ring pore openings, exhibit especially high ion-exchange capacities and strong affinity toward divalent metal ions [16,17]. Despite these advantages, the large-scale application of low-silica **CHA** zeolites (typically with Si/Al ≈ 2) remains limited by challenges associated with their synthesis [18,19,20,21,22,23,24,25]. Conventional methods often rely on costly **FAU**-type precursors, fluoride-containing mineralizers, or multistep procedures, resulting in complex processes and high production costs. Developing greener, simpler, and more economical synthesis routes for low-silica **CHA** zeolites therefore remains an important research goal.

Driven by the need for greener and more cost-effective synthesis routes, increasing attention has been directed toward the use of alternative silica and alumina sources for the production of **CHA**-type zeolites. Conventional methods typically rely on commercial reagents such as colloidal silica and aluminum hydroxide [26]. In contrast, waste-derived feedstocks, including fly ash [22,27] and rice husk ash [28], have emerged as promising alternatives. Despite these advances, the direct conversion of expanded perlite waste into high-purity **CHA** zeolite remains largely unreported.

In addition to synthesis, practical application requires evaluation under realistic industrial conditions. In industries such as electroplating, metallurgy, and oil refining, wastewater is often discharged at elevated temperatures (>50 °C) prior to cooling. Accordingly, it is essential to investigate the adsorption behavior of heavy metals over a wide temperature range. In this study, temperatures from ambient conditions (25 °C) to elevated levels (60–80 °C) were selected to simulate hot industrial effluents and to assess performance under realistic operating conditions.

Perlite is a naturally occurring volcanic glass composed predominantly of silica and alumina and typically contains 2–6% chemically bound water. Expanded perlite, the most widely used commercial form, is produced by rapidly heating raw perlite to approximately 870 °C, causing the bound water to vaporize and generate a lightweight, highly porous structure [29,30]. However, fine perlite particles often fail to expand efficiently during this process, leading to inferior products and the generation of large quantities of industrial waste [31,32]. This waste is difficult to manage and poses long-term environmental and land-use challenges. Although the recycling of perlite waste into zeolite-based composites has been explored [30,33,34], its direct utilization as a silica–alumina source for synthesizing high-purity, single-phase zeolites remains largely underexplored [35,36].

In this study, we report a simple, economical, and template-free one-pot strategy for the direct conversion of expanded perlite waste into high-purity **CHA** zeolite. The resulting material exhibits superior Pb^2+^ and Cd^2+^ removal performance compared with conventionally synthesized **CHA** zeolites, including ultrafast adsorption kinetics, high adsorption capacity, and excellent selectivity. This work provides a practical and sustainable pathway for perlite waste valorization while offering an efficient solution for heavy-metal wastewater treatment.

## 2. Results and Discussion

### 2.1. Characterization of Expanded Perlite Waste

X-ray fluorescence (XRF) analysis (Table S1) shows that SiO_2_ and Al_2_O_3_ together account for more than 89 wt.% of the expanded perlite waste, accompanied by minor amounts of Na, K, Ca, Fe, and Ti oxides.

**Figure 1:**
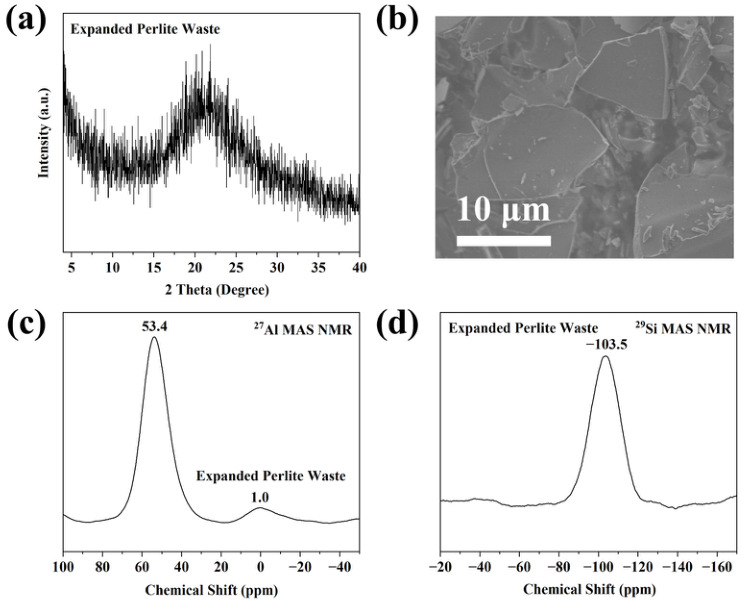
X-ray diffraction (XRD) pattern (**a**), Scanning electron microscopy (SEM) image (**b**), ^27^Al magic-angle spinning nuclear magnetic resonance (MAS NMR) spectrum (**c**), and ^29^Si magic-angle spinning nuclear magnetic resonance (MAS NMR) spectrum (**d**) of expanded perlite waste.

The X-ray diffraction (XRD) pattern (Figure 1a) displays a broad diffuse halo without sharp reflections, confirming the predominantly amorphous nature of the material. The scanning electron microscopy (SEM) image (Figure 1b) reveals irregular, sheet-like particles with an average size of ~10 μm.

The coordination environments of Si and Al were further investigated using ^27^Al and ^29^Si magic-angle spinning nuclear magnetic resonance (MAS NMR) spectroscopy (Figure 1c,d). The ^27^Al MAS NMR spectrum exhibits signals at 53.4 ppm and 1.0 ppm, corresponding to tetrahedrally coordinated framework Al and octahedrally coordinated extra-framework Al species, respectively [37]. The ^29^Si MAS NMR spectrum shows a dominant resonance at −103.5 ppm, assigned to Q^4^(1Al) silicon environments. These results confirm that expanded perlite waste provides a suitable amorphous silica–alumina source for zeolite synthesis.

### 2.2. Characterization of As-Synthesized CHA Zeolites

**Figure 2:**
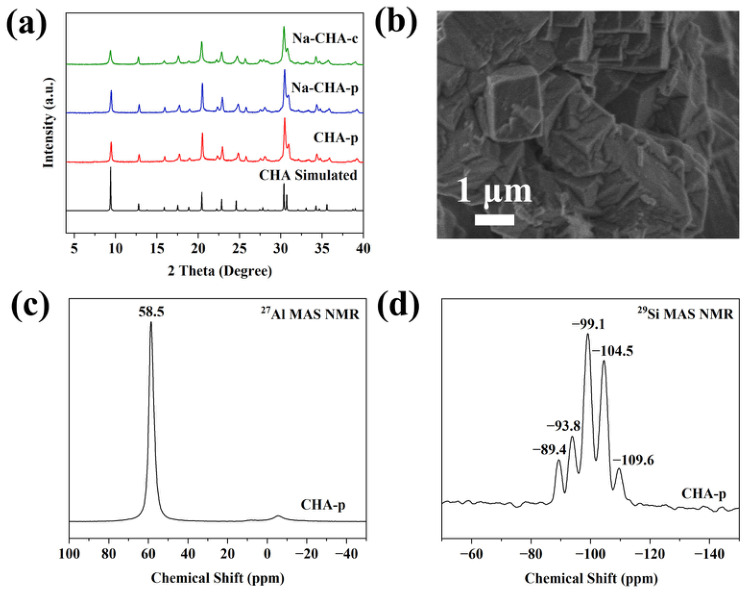
XRD patterns of the **CHA** zeolite synthesized from expanded perlite waste (CHA-p) and the reference sample synthesized from pure chemical reagents (CHA-c) after Na^+^ exchange (**a**), SEM image of CHA-p (**b**), ^27^Al MAS NMR spectrum (**c**), and ^29^Si MAS NMR spectrum (**d**) of CHA-p.

The **CHA** zeolite synthesized from expanded perlite waste is denoted as CHA-p, while the reference sample synthesized from pure chemical reagents is referred to as CHA-c. The XRD patterns of Na^+^-exchanged CHA-p (Na-CHA-p) and CHA-c (Na-CHA-c) (Figure 2a) closely match the simulated **CHA** pattern, confirming the formation of phase-pure **CHA** zeolite. No characteristic peaks associated with impurities from the raw perlite were detected, demonstrating the effectiveness of the synthesis protocol. The relative crystallinity of Na-CHA-p, determined by peak area integration, reaches 89.2% of that of Na-CHA-c. The SEM image (Figure 2b) shows aggregates of interconnected cuboid crystals with an average particle size of ~1.5 μm, consistent with reported morphologies of low-silica **CHA** zeolite [23].

Thermogravimetric (TG) analysis of Na-CHA-p and Na-CHA-c is presented in Figure S1. Both samples exhibit a major weight loss below 300 °C, attributed to the removal of weakly bound water (12.8 wt.% for Na-CHA-p and 15.6 wt.% for Na-CHA-c). A minor weight loss above 300 °C (0.6 wt.% and 1.0 wt.%, respectively) is associated with more strongly bound or framework-associated water [16]. These results provide a baseline for subsequent Pb^2+^ and Cd^2+^ adsorption studies.

By combining inductively coupled plasma optical emission spectrometry (ICP-OES) data (Table S2) with TG results, the unit-cell composition of CHA-p was determined as Na_1__.__4_K_11_Si_23__.__6_Al_12__.__4_O_72_·22.3H_2_O, corresponding to an Si/Al ratio of 1.9 and confirming the successful synthesis of low-silica **CHA** zeolite. The ^27^Al MAS NMR spectrum of CHA-p (Figure 2c) shows a dominant sharp resonance at 58.5 ppm and a weak, broad signal centered near 0 ppm. These signals are assigned to tetrahedral framework Al (Al^IV^) and minor octahedral extra-framework Al (Al^VI^) species, respectively [36]. Quantitative deconvolution using Lorentzian functions (Table S3) reveals that framework Al^IV^ accounts for 94.7% of the total aluminum signal, confirming the efficient incorporation of Al into the zeolite framework. The ^29^Si MAS NMR spectrum (Figure 2d) displays five resonances at −109.6, −104.5, −99.1, −93.8, and −89.4 ppm, corresponding to Si(0Al) to Si(4Al) environments, consistent with reported **CHA** structures [38]. The Si/Al ratio calculated from ^29^Si MAS NMR (2.2) is consistent with values obtained from ICP-OES (1.9) and XRF (1.9).

The N_2_ adsorption–desorption isotherms measured at 77 K for Ca^2+^-exchanged CHA-p (Ca-CHA-p) and CHA-c (Ca-CHA-c) are shown in Figure S2. Both samples exhibit steep uptake at low relative pressure (*P*/*P*_0_ < 0.05), characteristic of Type-I isotherms and indicative of well-developed microporosity. The specific surface area (*S*_BET_) was calculated using the multipoint Brunauer–Emmett–Teller (BET) method. The selected relative pressure ranges (0.0000–0.0198 for Ca-CHA-p and 0.0000–0.0230 for Ca-CHA-c) satisfy the Rouquerol criteria, ensuring the validity of the BET analysis. To avoid overestimation arising from interparticle capillary condensation, the total pore volume (*V*_total_) was determined at *P*/*P*_0_ ≤ 0.90, following established methodological recommendations [39]. The corresponding textural parameters are summarized in Table S4.

### 2.3. Crystallization Process of CHA-p

The crystallization behavior of CHA-p was monitored using XRD and SEM (Figures S3 and S4). Figure S3a compares XRD patterns collected at different crystallization times with the simulated **CHA** pattern, while Figure S3b shows the evolution of relative crystallinity. Corresponding morphological changes are presented in Figure S4.

During the first 6 h, the product remained amorphous, as evidenced by the absence of characteristic **CHA** reflections. After 12 h, weak **CHA** diffraction peaks appeared, corresponding to a relative crystallinity of 25.2%, accompanied by the formation of small cuboid crystals (Figures S3b and S4d). From 12 to 48 h, crystallinity increased rapidly, with the morphology evolving from loosely aggregated cuboids at 24 h to well-defined crystalline aggregates at 48 h (Figure S4e,f). Prolonging the crystallization time beyond 48 h did not further enhance crystallinity, indicating that 48 h is sufficient for the formation of highly crystalline CHA-p. The textural properties were further examined by a transmission electron microscope (TEM) (Figure S4g,h). The images show that the aggregates at both 24 h and 48 h are composed of dense crystalline subunits with well-defined edge contours. These features closely resemble the intergrown rhombohedral morphology. Notably, the TEM image of the sample crystallized at 48 h (Figure S4h) confirms the high crystallinity and structural integrity of the synthesized CHA-p, providing direct microscopic evidence that complements the XRD and SEM results.

### 2.4. Factors Affecting the Crystallization of CHA-p

The formation of phase-pure **CHA** zeolite is strongly influenced by gel alkalinity, Si/Al ratio, water content, and hydrothermal temperature. A gel composition of 0.16 Na_2_O:0.85 K_2_O:3.5 SiO_2_:Al_2_O_3_:111 H_2_O produced high-quality CHA-p. To accommodate the compositional complexity of expanded perlite waste, the synthesis parameters were systematically optimized. The optimal compositional window was identified as 0.16 Na_2_O:0.85 K_2_O:(3.5–4) SiO_2_:Al_2_O_3_:(111–131) H_2_O, as supported by Figure S5 and summarized in Table 1.

Hydrothermal synthesis conducted between 120 and 200 °C revealed that deviations from the optimal temperature range led to reduced phase purity. The optimal conditions are summarized in Run 1 (Table 1), while the conventional synthesis of CHA-c (Si/Al = 2.2) is provided for comparison in Run 8.

#### 2.4.1. Effect of K_2_O/SiO_2_ and Na_2_O/SiO_2_ Ratios

Previous studies have shown that K^+^ is essential for **CHA** formation under organic template-free conditions, whereas excessive K_2_O/SiO_2_ ratios favor the formation of **MER** zeolite [26]. In the present system, K_2_O/SiO_2_ ratios of 0.41 and 0.47 resulted in mixed **CHA**/**MER** phases (Figure S5a; Table 1, Runs 2 and 3). Reducing this ratio to 0.24 yielded phase-pure CHA-p (Figure S5a; Table 1, Run 1).

Although both K^+^ and Na^+^ are commonly used in **CHA** synthesis [40], excessive Na^+^ increases gel alkalinity and promotes **MER** formation. This effect was observed at a Na_2_O/SiO_2_ ratio of 0.13 (Figure S5b; Table 1, Run 4). Notably, phase-pure CHA-p was obtained without external Na^+^ addition, relying solely on sodium inherently present in the perlite waste (Figure S5b; Table 1, Run 1).

#### 2.4.2. Effect of SiO_2_/Al_2_O_3_ Ratio

To obtain low-silica **CHA**, the SiO_2_/Al_2_O_3_ ratio was systematically varied. Ratios below 3.5 failed to yield **CHA**, whereas ratios between 3.5 and 4 produced phase-pure **CHA** with an Si/Al ratio of 1.9–2.2 (Figure S5c; Table 1, Runs 1 and 5). An SiO_2_/Al_2_O_3_ ratio of 3.5 was selected as optimal, enabling the synthesis of low-silica **CHA** without compromising crystallinity.

#### 2.4.3. Effect of H_2_O/SiO_2_ Ratio

As shown in Figure S5d, an excessively high H_2_O/SiO_2_ ratio (43.1) suppressed **CHA** formation. Reducing this ratio to 31.7–37.4 enabled the formation of phase-pure CHA-p (Figure S5d; Table 1, Runs 1 and 6). To minimize dilution effects and maximize product yield, the lowest effective ratio (31.7) was selected as optimal.

#### 2.4.4. Effect of Crystallization Temperature

The effect of crystallization temperature was evaluated between 170 and 180 °C. Both samples synthesized at 170 °C and 180 °C were phase-pure CHA-p, with relative crystallinity of 100% at 180 °C and 82.6% at 170 °C (Figure S5e; Table 1, Runs 1 and 7). This result indicates that increasing the synthesis temperature promotes more complete crystallization of the perlite-derived aluminosilicate gel. Accordingly, 180 °C was selected as the optimal crystallization temperature.

### 2.5. Removal of Pb^2+^ and Cd^2+^

Prior to adsorption experiments, CHA-p and CHA-c were converted to their Na^+^ forms (denoted Na-CHA-p and Na-CHA-c, respectively) via ion exchange. Na-CHA-c was used as the reference material.

#### 2.5.1. Influence of Adsorbent Dosage

**Figure 3:**
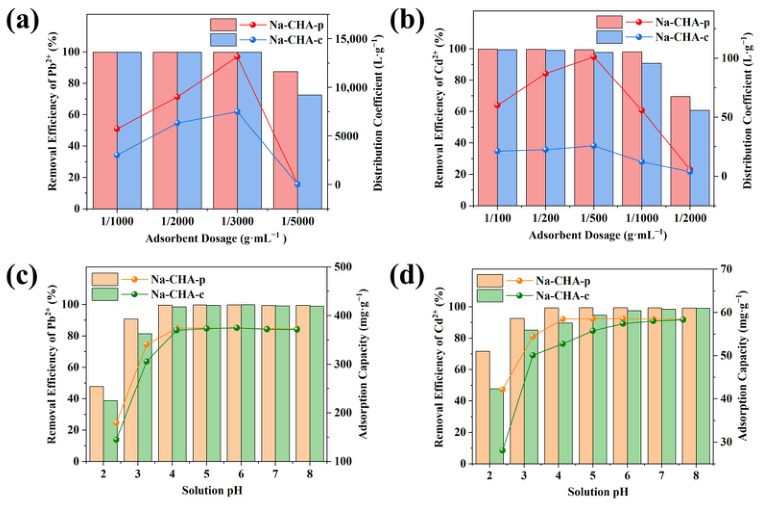
Influence of adsorbent dosage on the adsorption of Pb^2+^ (**a**) and Cd^2+^ (**b**) by Na^+^-exchanged CHA-p (Na-CHA-p) and Na^+^-exchanged CHA-c (Na-CHA-c); influence of initial solution pH on the adsorption of Pb^2+^ (**c**) and Cd^2+^ (**d**) by Na-CHA-p and Na-CHA-c.

The influence of adsorbent dosage on Pb^2+^ removal was first evaluated using a Pb^2+^ solution with an initial concentration of 100 mg·L^−1^ (15 mL). As shown in Figure 3a, both the removal efficiency and the distribution coefficient (*K*_d_) were strongly dependent on the adsorbent dosage. At a dosage of 1/1000 g·mL^−1^, both Na-CHA-p and Na-CHA-c achieved Pb^2+^ removal efficiencies of 99.9%. This high performance was maintained at lower dosages of 1/2000 and 1/3000 g·mL^−1^.

The maximum *K*_d_ value was observed at a dosage of 1/3000 g·mL^−1^, where Na-CHA-p (1.3 × 10^4^ L·g^−1^) significantly outperformed Na-CHA-c (7.5 × 10^3^ L·g^−1^). The lower adsorption capacity of Na-CHA-c is attributed to its lower alumina content, which results in fewer available ion-exchange sites [41]. This explains why Na-CHA-p exhibits superior Pb^2+^ and Cd^2+^ adsorption despite its lower surface area and pore volume (Table S4). Further reduction of the dosage to 1/5000 g·mL^−1^ led to a pronounced decline in Pb^2+^ removal efficiency, decreasing to 87.6% for Na-CHA-p and 72.7% for Na-CHA-c. Accordingly, a dosage of 1/3000 g·mL^−1^ was selected as optimal for Pb^2+^ removal using Na-CHA-p.

For Cd^2+^ removal (100 mg·L^−1^, 10 mL), the optimal dosage was determined to be 1/500 g·mL^−1^ (Figure 3b). At this dosage, Na-CHA-p achieved a Cd^2+^ removal efficiency of 99.4% and a *K*_d_ value of 101.1 L·g^−1^, significantly higher than those of Na-CHA-c (97.8% and 25.7 L·g^−1^). These results clearly demonstrate the superior adsorption efficiency of Na-CHA-p for both Pb^2+^ and Cd^2+^ at optimized dosages.

The superior adsorption performance of Na-CHA-p (Figure 3) arises from its well-defined, phase-pure **CHA** framework featuring 8-ring pore openings, cubic crystals of ~1.5 μm, as well as the high microporous surface area of 456.58 m^2^·g^−1^ of Ca-CHA-p (Table S4). The preferential adsorption of Pb^2+^ over Cd^2+^ is primarily governed by its lower hydration energy, which facilitates dehydration and enhances diffusion into the zeolitic micropores [42].

#### 2.5.2. Influence of Initial pH

The influence of initial solution pH on Pb^2+^ and Cd^2+^ removal by Na-CHA-p and Na-CHA-c was investigated over a pH range of 2–8. Based on the solubility product constants (*K*_sp_), precipitation of Pb^2+^ and Cd^2+^ at a concentration of 100 mg·L^−1^ would occur at pH values above 8.6 and 8.5, respectively. All experiments were therefore conducted below these thresholds to eliminate contributions from metal hydroxide precipitation.

As shown in Figure 3c, Pb^2+^ removal was strongly suppressed at pH 2, with removal efficiencies of 47.9% for Na-CHA-p and 38.7% for Na-CHA-c. This behavior is attributed to strong competition between H_3_O^+^ and Pb^2+^ for ion-exchange sites under highly acidic conditions [15]. Increasing the pH to 3 resulted in a sharp increase in Pb^2+^ removal, reaching 90.8% for Na-CHA-p and 81.5% for Na-CHA-c. At pH values between 4 and 8, both materials exhibited consistently high removal efficiencies, exceeding 99.5% for Na-CHA-p and 98.6% for Na-CHA-c. The adsorption capacity followed a similar trend, reflecting the diminished competitive effect of protons at higher pH.

Cd^2+^ adsorption showed a comparable pH dependence (Figure 3d). At pH values between 4 and 8, Na-CHA-p maintained Cd^2+^ removal efficiencies above 99.3%, whereas Na-CHA-c achieved efficiencies above 89.7%. Across the entire pH range examined, Na-CHA-p consistently outperformed Na-CHA-c. These results demonstrate that Na-CHA-p provides stable and efficient Pb^2+^ and Cd^2+^ removal under environmentally relevant pH conditions, highlighting its suitability for practical wastewater treatment.

#### 2.5.3. Selectivity Toward Pb^2+^ and Cd^2+^

In practical wastewater systems, Pb^2+^ and Cd^2+^ coexist with other heavy metals and with high concentrations of common cations such as Na^+^, K^+^, Ca^2+^, and Mg^2+^ [43]. Because zeolites preferentially adsorb cations with a high charge density, small hydrated radius, and low hydration energy [44], competitive adsorption experiments were conducted to assess the selectivity of Na-CHA-p under multi-ion conditions. Parallel experiments using Na-CHA-c were performed for comparison.

**Figure 4:**
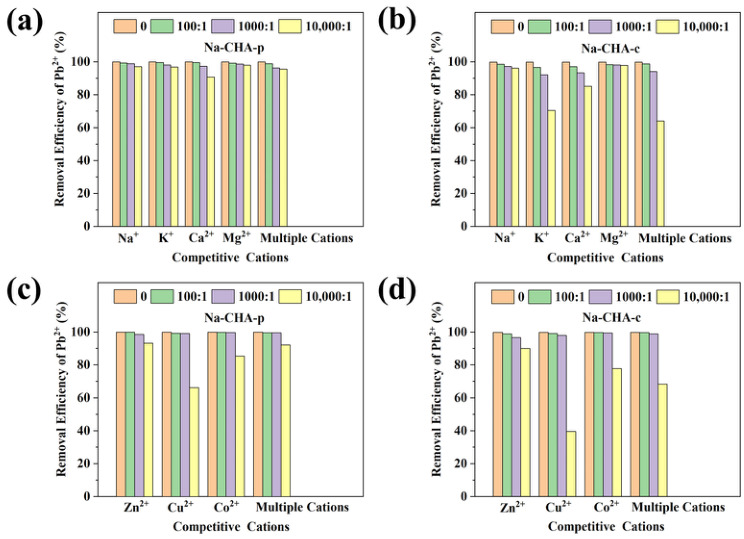
Influence of Na^+^, K^+^, Ca^2+^, and Mg^2+^ cations and their mixture on Pb^2+^ adsorption by Na-CHA-p (**a**) and Na-CHA-c (**b**); influence of Zn^2+^, Cu^2+^, Co^2+^ cations and their mixture on Pb^2+^ adsorption by Na-CHA-p (**c**) and Na-CHA-c (**d**).

For Pb^2+^ adsorption in the presence of competitive alkali and alkaline-earth cations, the molar ratio of competitive ions (M^n+^) to Pb^2+^ was varied from 100:1 to 10,000:1 (Figure 4a,b). At M^n+^/Pb^2+^ ratios of 100:1 and 1000:1, Na-CHA-p retained Pb^2+^ removal efficiencies above 96.3%, whereas Na-CHA-c showed slightly lower efficiencies (>92.1%). This superior performance of Na-CHA-p is primarily attributed to the lower hydration energy of Pb^2+^ relative to the competitive cations [45]. When the ratio increased to 10,000:1, Pb^2+^ removal decreased noticeably, particularly in the presence of Ca^2+^, where the removal efficiency dropped to 90.7%. This pronounced inhibition arises from the high charge density of Ca^2+^ combined with its overwhelming concentration [16].

The selectivity of Na-CHA-p toward Pb^2+^ was further evaluated in the presence of competitive divalent heavy metal ions (Zn^2+^, Cu^2+^, Co^2+^, or their mixture). As shown in Figure 4c,d, Pb^2+^ removal efficiencies remained above 98.5% for Na-CHA-p and 96.7% for Na-CHA-c at M^2+^/Pb^2+^ ratios ranging from 100:1 to 1000:1. These results confirm the strong preference of **CHA** zeolites—particularly Na-CHA-p—for Pb^2+^ in complex heavy-metal systems, again reflecting its relatively low hydration energy [45]. At a ratio of 10,000:1, Pb^2+^ removal declined substantially, with Cu^2+^ exerting the strongest inhibitory effect due to its small hydrated radius [15].

The selectivity toward Cd^2+^ was examined under analogous conditions (Figure S6). In the presence of common cations at an M^n+^/Cd^2+^ ratio of 100:1, Na-CHA-p maintained Cd^2+^ removal efficiencies above 95.5%, significantly higher than those of Na-CHA-c (>69.8%) (Figure S6a,b). Increasing the ratio to 1000:1 led to a marked decline in Cd^2+^ removal, particularly in the presence of Ca^2+^, owing to its high charge density and competitive advantage [16]. In systems containing multiple heavy-metal ions (Figure S6c,d), Cu^2+^ again exerted the strongest inhibitory effect on Cd^2+^ adsorption, consistent with its small hydrated radius. Overall, Na-CHA-p exhibits superior selectivity for Cd^2+^ compared with Na-CHA-c, although its selectivity is lower than that observed for Pb^2+^, reflecting the more favorable hydration properties of Pb^2+^.

#### 2.5.4. Adsorption Kinetics

**Figure 5:**
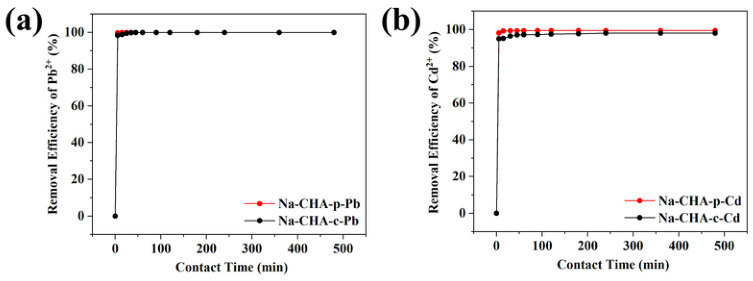
Adsorption kinetics of Pb^2+^ (**a**) and Cd^2+^ (**b**) on Na-CHA-p and Na-CHA-c.

Adsorption mechanisms of heavy metals on zeolites are commonly inferred from kinetic and isotherm analyses [46]. The time-dependent adsorption behavior of Pb^2+^ and Cd^2+^ on Na-CHA-p and Na-CHA-c is shown in Figure 5.

As shown in Figure 5a, Na-CHA-p exhibited extremely rapid Pb^2+^ uptake, achieving 99.7% removal within the first 5 min. Only marginal increases were observed thereafter, indicating that adsorption equilibrium was rapidly attained. Kinetic data were fitted using pseudo-first-order and pseudo-second-order models (Figure S7a,b). As summarized in Table S5, the pseudo-second-order model provided a significantly better fit (*R*^2^ = 0.999) than the pseudo-first-order model (*R*^2^ = 0.795), suggesting that Pb^2+^ adsorption is dominated by a chemisorption mechanism involving ion exchange [47]. Na-CHA-c displayed a similar kinetic profile, indicating an analogous adsorption mechanism.

Cd^2+^ adsorption on Na-CHA-p also reached equilibrium within 5 min (Figure 5b). Kinetic modeling again confirmed that the pseudo-second-order model best described the experimental data (*R*^2^ = 0.999) (Figure S7c,d and Table S5), confirming that Cd^2+^ uptake is likewise governed by chemisorption. These results highlight the exceptionally fast adsorption kinetics of Na-CHA-p, which are highly advantageous for practical water treatment applications.

#### 2.5.5. Adsorption Isotherms

**Figure 6:**
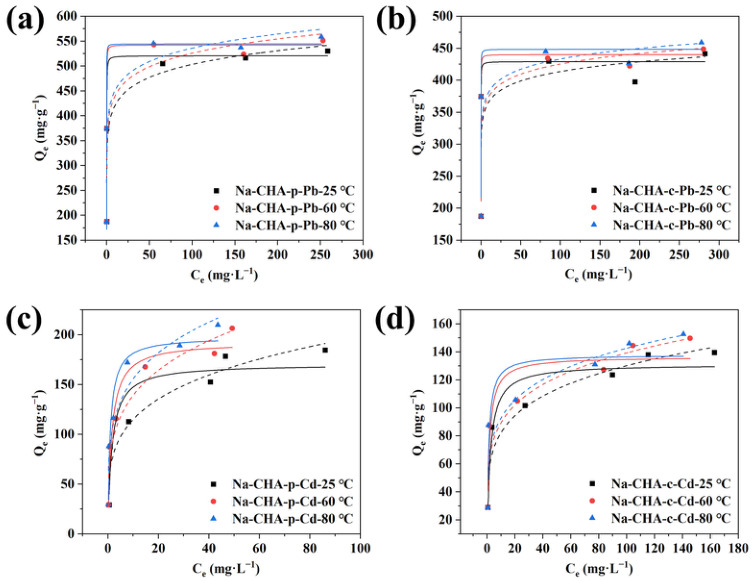
Adsorption isotherms of Pb^2+^ on Na-CHA-p (**a**) and Na-CHA-c (**b**), and of Cd^2+^ on Na-CHA-p (**c**) and Na-CHA-c (**d**). Solid and dashed lines represent Langmuir and Freundlich fittings, respectively.

To assess the feasibility of Na-CHA-p for treating hot industrial effluents, adsorption isotherms for Pb^2+^ and Cd^2+^ were measured at 25, 60, and 80 °C. The equilibrium adsorption behavior was analyzed using the Langmuir and Freundlich isotherm models. The experimental isotherm data (Figure 6), along with their linear fittings (Figures S8 and S9), demonstrate that adsorption of both metal ions is well described by the Langmuir model, indicating monolayer adsorption on a homogeneous distribution of active sites [48]. The corresponding fitting parameters are summarized in Tables S6 and S7.

The maximum adsorption capacities (*Q*_m_) increased progressively with temperature from 25 °C to 80 °C, confirming the endothermic nature of the adsorption process. At 80 °C, which is representative of hot industrial effluent, Na-CHA-p exhibited exceptional high capacities of 555.6 mg·g^−1^ for Pb^2+^ and 211.0 mg·g^−1^ for Cd^2+^, which are substantially higher than those of Na-CHA-c (450.5 mg·g^−1^ for Pb^2+^ and 153.1 mg·g^−1^ for Cd^2+^). Comparison with previously reported adsorbents (Table 2 and Table 3) demonstrates the outstanding adsorption performance of Na-CHA-p for both metal ions.

Based on elemental compositions obtained from XRF analysis (Tables S1 and S8), the theoretical maximum adsorption capacities of Na-CHA-p were calculated to be 638.4 mg·g^−1^ for Pb^2+^ and 346.3 mg·g^−1^ for Cd^2+^. These values exceed the experimentally observed capacities, indicating that not all ion-exchange sites are fully utilized under the tested conditions and suggesting further potential for performance optimization.

#### 2.5.6. Reusability

**Figure 7:**
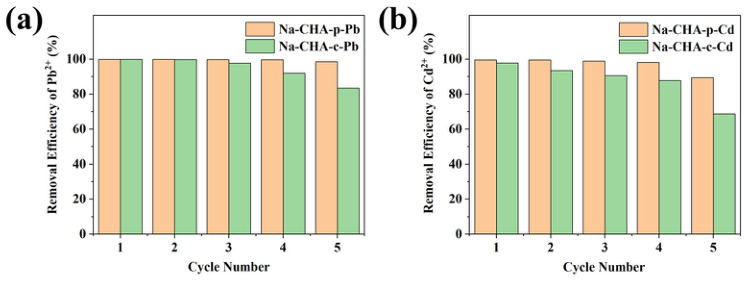
Reusability of Na-CHA-p and Na-CHA-c for Pb^2+^ (**a**) and Cd^2+^ (**b**) removal.

The reusability of Na-CHA-p and Na-CHA-c was evaluated over five consecutive adsorption–desorption cycles (Figure 7).

For Pb^2+^ removal, Na-CHA-p maintained a removal efficiency above 98.5% throughout all five cycles. In contrast, Na-CHA-c retained efficiencies above 97.7% only during the first three cycles, followed by a decline to 91.9% in the fourth cycle. This behavior indicates inferior cycling stability of Na-CHA-c. The observed decrease is attributed to incomplete Pb^2+^ desorption and progressive structural deterioration. XRD analysis after cycling (Figure S10) confirms that the framework of Na-CHA-p remains largely well preserved, whereas Na-CHA-c undergoes more pronounced degradation.

For Cd^2+^ removal, Na-CHA-p sustained removal efficiencies above 98.2% over the first four cycles, with a decrease to 89.5% in the fifth cycle. In contrast, Na-CHA-c showed a continuous decline in performance starting from the second cycle. Consistent with these results, XRD analysis confirms that the framework structure of Na-CHA-p remains largely intact after repeated cycling.

### 2.6. Leachability and Retention Mechanism of Spent Adsorbents

As shown in Table S9, the leaching concentrations of Pb^2+^ from exhausted Na-CHA-p and Na-CHA-c were 0.21 and 0.48 mg·L^−1^, respectively, while those of Cd^2+^ were 0.11 and 0.25 mg·L^−1^. All values are well below the US EPA regulatory limits for hazardous waste (5.0 mg·L^−1^ for Pb and 1.0 mg·L^−1^ for Cd) [66], indicating a low risk of secondary contamination under landfill conditions. The low leachability is attributed to the strong retention of Pb^2+^ and Cd^2+^ at ion-exchange sites, coupled with the constrained pore openings of the **CHA** framework, which limit outward diffusion of the entrapped ions [67].

## 3. Materials and Methods

### 3.1. Materials

Expanded perlite waste was obtained from Changchun Songbinlinyun Building Materials Co., Ltd., Changchun, China. The following reagents were used as received: pseudo-boehmite (Sasol, Hamburg, Germany, 72.7 wt.%), NaCl (Tianjin Guangfu Fine Chemical Research Institute, Tianjin, China), NaOH (Tianjin Fuchen Chemical Reagents Factory, Tianjin, China, NaOH ≥ 96.0%), KOH (Tianjin Guangfu Fine Chemical Research Institute, KOH ≥ 85.0%), Pb(NO_3_)_2_ (Shanghai Aladdin Biochemical Technology Co., Ltd., Shanghai, China), NaNO_3_ (Beijing Chemical Works, Beijing, China), KNO_3_ (Sinopharm Chemical Reagent Co., Ltd., Shanghai, China), Ca(NO_3_)_2_ (Tianjin Fuchen Chemical Reagents Factory), Mg(NO_3_)_2_ (Sinopharm Chemical Reagent Co., Ltd.), HNO_3_ (Beijing Chemical Works), CdCl_2_·2.5H_2_O (Shanghai Macklin Biochemical Technology Co., Ltd., Shanghai, China), KCl (Tianjin Fuchen Chemical Reagents Factory), CaCl_2_ (Tianjin Guangfu Fine Chemical Research Institute), MgCl_2_ (Shanghai Aladdin Biochemical Technology Co., Ltd.), Zn(NO_3_)_2_·6H_2_O (Shanghai Macklin Biochemical Technology Co., Ltd.), Cu(NO_3_)_2_·3H_2_O (Shanghai Macklin Biochemical Technology Co., Ltd.), Co(NO_3_)_2_·6H_2_O (Shanghai Macklin Biochemical Technology Co., Ltd.), and HCl (Beijing Chemical Reagent Research Institute Co., Ltd., Beijing, China). Ludox AS-40 (40 wt.%, suspension in water) was purchased from Aldrich (Saint Louis, MO, USA).

### 3.2. Synthesis of CHA Zeolite

#### 3.2.1. Synthesis of CHA-p Zeolite

Zeolite CHA-p was synthesized from an initial gel with a molar composition of 0.16 Na_2_O:0.85 K_2_O:3.5 SiO_2_:Al_2_O_3_:111 H_2_O. To prepare the gel, 1.84 g of KOH was dissolved in 40 mL of deionized water. The Na_2_O component was supplied from the expanded perlite waste. Subsequently, 1.78 g of pseudo-boehmite and 5.52 g of expanded perlite waste were added while stirring. The mixture was stirred for 4 h to ensure homogeneity, then transferred to a Teflon-lined stainless-steel autoclave and heated at 180 °C for 48 h. The resulting solid was collected by filtration, thoroughly washed with deionized water until the pH was below 8, and dried at 80 °C.

#### 3.2.2. Synthesis of CHA-c Zeolite

For comparison, zeolite CHA-c was synthesized from pure chemical raw materials according to a previous report [17], with a molar composition of 0.092 Na_2_O:Al_2_O_3_:3.99 SiO_2_:1.067 K_2_O:171 H_2_O. In a typical synthesis, 0.096 g of NaOH and 1.83 g of KOH were dissolved in 35 mL of deionized water. Then, 1.82 g of pseudo-boehmite and 7.78 g of Ludox AS-40 were slowly added while stirring. The mixture was stirred for 3 h to ensure homogeneity, then transferred to a Teflon-lined stainless-steel autoclave and heated at 160 °C for 96 h. The product was treated using the same method as described in Section 3.2.1.

### 3.3. Na^+^ Modification of CHA-p and CHA-c

CHA-p and CHA-c were ion-exchanged with 1.0 mol·L^−1^ NaCl solution at a solid-to-liquid ratio of 20 g·L^−1^. The suspensions were stirred at 60 °C for 3 h. Next, the solid was filtered and washed with deionized water until the pH was below 8. This process was repeated twice more (three times in total). Finally, the ion-exchanged product was dried at 80 °C. The Na^+^-exchanged samples are referred to as Na-CHA-p and Na-CHA-c, respectively, and were used in subsequent Pb^2+^ and Cd^2+^ adsorption experiments.

### 3.4. Pb^2+^ and Cd^2+^ Adsorption Experiments

To evaluate adsorption performance, batch experiments were conducted for Pb^2+^ and Cd^2+^ removal using Na-CHA-p and Na-CHA-c at 25 °C (ambient condition), 60 °C, and 80 °C (industrial condition). For Pb^2+^ removal, 5 mg of zeolite was dispersed into 15 mL of solution with various Pb^2+^ concentrations. For Cd^2+^ removal, 20 mg of zeolite was dispersed into 10 mL of solution with various Cd^2+^ concentrations. The pH of the Pb^2+^ solution was adjusted using 1.0 mol·L^−1^ KOH or 1.0 mol·L^−1^ HNO_3_, while the pH of the Cd^2+^ solution was adjusted using 1.0 mol·L^−1^ KOH or 1.0 mol·L^−1^ HCl. The mixtures were stirred for 8 h. The solution was then filtered using a 0.22 μm polyethersulfone (PES) membrane filter, and the concentrations of Pb^2+^ or Cd^2+^ in the filtrate were measured by ICP-OES. The physically adsorbed water content in zeolite **CHA** was determined by TG analysis, and adsorption capacities were calculated based on the dry mass of the zeolite.

The removal efficiency *R* (%), equilibrium adsorption capacity *Q*_e_ (mg⋅g^−1^), and distribution coefficient *K*_d_ (L⋅g^−1^) were calculated as:

(1) $$\begin{array}{c} R\left(\%\right)=\frac{C_{i}-C_{e}}{C_{i}}\times 100\%, \end{array}$$

(2) $$\begin{array}{c} Q_{e}=\left(C_{i}-C_{e}\right)\times \frac{V}{m}\; \end{array}$$

and

(3) $$\begin{array}{c} K_{d}=\frac{C_{i}-C_{e}}{C_{e}}\times \frac{V}{m}\;, \end{array}$$

where *C*_i_ and *C*_e_ (mg⋅L^−1^) are the initial and equilibrium concentrations of Pb^2+^ or Cd^2+^, respectively; *V* (L) is the solution volume; and *m* (g) is the dry zeolite mass. Na-CHA-c was employed as a reference material.

#### 3.4.1. Influence of Adsorbent Dosage

The influence of the adsorbent dosage on Pb^2+^ removal was examined using solid-to-liquid ratios of 1/1000, 1/2000, 1/3000 and 1/5000 (g⋅mL^−1^), while that on Cd^2+^ removal was examined using solid-to-liquid ratios of 1/100, 1/200, 1/500, 1/1000 and 1/2000 (g⋅mL^−1^). The initial Pb^2+^ or Cd^2+^ concentration was 100 mg⋅L^−1^, and the initial pH was adjusted to 6.0. The contact time was 8 h under stirring.

#### 3.4.2. Influence of Initial pH

The influence of pH on Pb^2+^ or Cd^2+^ removal was investigated within a pH range from 2 to 8. The adsorbent dosage was 1/3000 (g⋅mL^−1^) for Pb^2+^ removal and 1/500 (g⋅mL^−1^) for Cd^2+^ removal. The initial Pb^2+^ or Cd^2+^ concentration was 100 mg⋅L^−1^, with a contact time of 8 h under stirring.

#### 3.4.3. Selectivity Toward Pb^2+^ and Cd^2+^

For Pb^2+^ removal, the selectivity of Na-CHA-p and Na-CHA-c was tested using 5 mg·L^−1^ Pb^2+^ solutions at 25 °C containing Na^+^, K^+^, Ca^2+^, Mg^2+^, or a mixture at molar ratios of M^n+^/Pb^2+^ = 0, 100:1, 1000:1 and 10,000:1. For selectivity tests involving other heavy metal cations, Na-CHA-p and Na-CHA-c were added to 5 mg·L^−1^ Pb^2+^ solutions at 25 °C containing Zn^2+^, Cu^2+^, Co^2+^, or a mixture at molar ratios of M^2+^/Pb^2+^ = 0, 100:1, 1000:1 and 10,000:1. For mixed-ion systems, the cations were present in an equimolar ratio (1:1:1:1). The pH of the mixed solutions was set as 6.0, and the contact time was 8 h under stirring.

For Cd^2+^ removal, the selectivity of Na-CHA-p and Na-CHA-c was tested using 5 mg·L^−1^ Cd^2+^ solutions at 25 °C containing Na^+^, K^+^, Ca^2+^, Mg^2+^, or a mixture at molar ratios of M^n+^/Cd^2+^ = 0, 100:1 and 1000:1. For selectivity tests with other heavy metal cations, Na-CHA-p and Na-CHA-c were added to 5 mg·L^−1^ Cd^2+^ solutions at 25 °C containing Zn^2+^, Cu^2+^, Co^2+^, or a mixture at molar ratios of M^2+^/Cd^2+^ = 0, 10:1 and 100:1. For mixed-ion systems, the cations were present in an equimolar ratio (1:1:1:1). The pH of the mixed solutions was set as 6.0, and the contact time was 8 h under stirring.

#### 3.4.4. Adsorption Kinetics

The influence of contact time on Pb^2+^ or Cd^2+^ removal was studied from 5 min to 480 min with an initial pH of 6.0. The adsorbent dosage was 1/3000 (g⋅mL^−1^) for Pb^2+^ removal and 1/500 (g⋅mL^−1^) for Cd^2+^ removal, and the initial concentration of both ions was 100 mg⋅L^−1^.

The kinetic data were fitted using pseudo-first-order and pseudo-second-order models:

Pseudo-first-order model [68]:

(4) $$\begin{array}{c} Q_{t}=Q_{e}\left(1-e^{-K_{1}t}\right)\;, \end{array}$$

where *Q*_t_ (mg⋅g^−1^) is the adsorption amount at the adsorption time of *t* (min), *Q*_e_ (mg⋅g^−1^) is the equilibrium adsorption capacity, and *K*_1_ (min^−1^) is the rate constant.

Pseudo-second-order model [69,70]:

(5) $$\begin{array}{c} Q_{t}=\frac{K_{2}Q_{e}^{2}t}{1+K_{2}Q_{e}t}\;, \end{array}$$

where *K*_2_ (g⋅mg^−1^⋅min^−1^) is the rate constant, and other parameters are defined as above.

#### 3.4.5. Adsorption Isotherms

Adsorption isotherms were measured at 25 °C, 60 °C, and 80 °C with initial Pb^2+^ or Cd^2+^ concentrations ranging from 50 to 400 mg⋅L^−1^. The adsorbent dosage was 1/3000 (g⋅mL^−1^) for Pb^2+^ removal and 1/500 (g⋅mL^−1^) for Cd^2+^ removal. The initial pH was set as 6.0, with a contact time of 8 h.

The data were fitted using the Langmuir and Freundlich models:

Langmuir model [48]:

(6) $$\begin{array}{c} Q_{e}=\frac{Q_{m}K_{L}C_{e}}{1+K_{L}C_{e}}\;, \end{array}$$

where *Q*_m_ (mg·g^−1^) is the maximum adsorption capacity and *K*_L_ (L·mg^−1^) is the Langmuir constant.

Freundlich model [71]:

(7) $$\begin{array}{c} Q_{e}=K_{F}C_{e}^{\frac{1}{n}}\;, \end{array}$$

where *K*_F_ (mg^1−1/n^·L^1/n^·g^−1^) and *n* are Freundlich constants related to adsorption capacity and intensity. The model fits were evaluated by linear regression.

#### 3.4.6. Reusability

The reusability of Na-CHA-p and Na-CHA-c for Pb^2+^ and Cd^2+^ removal was systematically evaluated. For Pb^2+^ adsorption, a solid-to-liquid ratio of 1/3000 g⋅mL^−1^ was used, whereas for Cd^2+^ adsorption, the ratio was 1/500 g⋅mL^−1^. All experiments were conducted at initial pH of 6 with a contact time of 8 h. After each adsorption cycle, the spent adsorbents were regenerated by immersion in 1 mol⋅L^−1^ NaCl solution for 12 h, followed by thorough washing with deionized water. The adsorption–regeneration process was repeated for five consecutive cycles.

### 3.5. Leaching Tests

The leaching stability of the spent adsorbents was evaluated using the Toxicity Characteristic Leaching Procedure (TCLP) method [72]. Metal-loaded samples were mixed with acetic acid extraction fluid (pH 2.88) at a liquid-to-solid ratio of 20 mL·g^−1^ and agitated for 18 h. The concentrations of Pb^2+^ and Cd^2+^ in the resulting leachates were quantified by ICP-OES.

### 3.6. Characterization

Powder X-ray diffraction (PXRD) patterns were collected over 2θ = 4–40° using a Rigaku (Tokyo, Japan) D/Max 2550 diffractometer with Cu Kα radiation (λ = 1.5418 Å) and a step size of 0.02°. The relative crystallinity of CHA-p was calculated as:

(8) $$\begin{array}{c} Relative\;Crystallinity=\frac{\sum A_{sample}}{\sum A_{reference}}\times 100\%\;, \end{array}$$

where A is the integrated area of a characteristic diffraction peak. The calculation was based on the **CHA** reflections located at approximately 2θ = 9.4°, 12.8°, 15.9°, 17.6°, 20.4°, 22.9°, 24.7°, 30.4°, 34.3°, and 35.8°.

The chemical composition of expanded perlite waste and Na-CHA-p was analyzed by XRF using a PANalytical (Worcestershire, UK) Axios Advanced X-ray fluorescence spectrometer. Morphologies were examined by SEM using a Jeol JSM-7800F microscope (Peabody, MA, USA). TEM images were obtained with the electron microscope Tecnai F20 (Hillsboro, OR, USA). TG analysis was conducted in air on a Q500 analyzer (New Castle, DE, USA) with a heating rate of 10 °C⋅min^−1^ from ambient temperature to 800 °C. The Si/Al ratio and the concentrations of Pb^2+^ and Cd^2+^ were determined by ICP-OES using a Thermo Scientific (Waltham, MA, USA) iCAP 7600 DUO instrument. ^27^Al and ^29^Si MAS NMR spectra were acquired on a Bruker (Billerica, MA, USA) Avance NEO spectrometer at 14.09 T to probe the local atomic environments. The Si/Al ratio was calculated from the NMR data using the following formula:

(9) $$\begin{array}{c} {\left(\frac{Si}{Al}\right)}_{NMR}=\frac{\sum_{n=0}^{n=4}I_{Si\left(nAl\right)}}{\sum_{n=0}^{n=4}{0.25\times n\times I}_{Si\left(nAl\right)}}. \end{array}$$

N_2_ adsorption–desorption isotherms were measured at 77 K using a BSD-660M analyzer (Beijing, China). Prior to analysis, the samples were degassed at 573 K for at least 8 h. Surface areas and pore volumes were calculated using the BET and t-plot methods, respectively. The total pore volume was determined at *P*/*P*_0_ = 0.90.

## 4. Conclusions

In this study, a low-silica **CHA** zeolite was synthesized directly from expanded perlite waste via a one-pot, template-free hydrothermal strategy, providing a sustainable route for waste valorization. After Na^+^ exchange, the resulting Na-CHA-p exhibited exceptional performance for Pb^2+^ and Cd^2+^ removal from aqueous solutions. At 25 °C and an initial concentration of 100 mg·L^−1^, ultrafast adsorption was achieved, reaching equilibrium within 5 min, with removal efficiencies of 99.9% for Pb^2+^ and 99.4% for Cd^2+^ (solid-to-liquid ratio: 1/3000 g⋅mL^−1^ for Pb^2+^ and 1/500 g⋅mL^−1^ for Cd^2+^). The material delivered high adsorption capacities of 555.6 mg·g^−1^ for Pb^2+^ and 211.0 mg·g^−1^ for Cd^2+^, together with strong selectivity in the presence of competing cations. Notably, Na-CHA-p demonstrated excellent durability, maintaining >98.5% Pb^2+^ removal over five cycles and >98.2% Cd^2+^ removal over four cycles. Leaching tests confirmed the robust immobilization of heavy metals, with Pb^2+^ and Cd^2+^ concentrations remaining well below EPA regulatory limits under acidic conditions, highlighting the environmental safety of the spent adsorbent. Overall, this study establishes a scalable and sustainable strategy for converting industrial waste into a high-performance zeolitic adsorbent, offering significant potential for rapid, selective, and practical heavy-metal remediation in complex aqueous systems.

## Figures and Tables

**Table 1:** Synthesis conditions and elemental compositions of CHA samples.

Run	Sample ^1^	K_2_O/SiO_2_	Na_2_O/SiO_2_	SiO_2_/Al_2_O_3_	H_2_O/SiO_2_	Temperature	Crystallization Phase	Si/Al ^2^	Si/Al ^3^
1	CHA-p	0.24	0.05	3.5	31.7	180 °C	**CHA**	1.9	1.9
2	CHA-p-0.41	0.41	0.05	3.5	31.7	180 °C	**CHA + MER**		
3	CHA-p-0.47	0.47	0.05	3.5	31.7	180 °C	**CHA + MER**		
4	CHA-p-0.13	0.24	0.13	3.5	31.7	180 °C	**CHA + MER**		
5	CHA-p-4	0.24	0.05	4	31.7	180 °C	**CHA**	2.2	
6	CHA-p-37.4	0.24	0.05	3.5	37.4	180 °C	**CHA**	2.0	
7	CHA-p-170	0.24	0.05	3.5	31.7	170 °C	**CHA**	2.0	
8	CHA-c	0.27	0.02	3.99	42.9	160 °C	**CHA**	2.2	

^1^ Sample names reflect key synthetic parameters (K_2_O/SiO_2_, Na_2_O/SiO_2_, SiO_2_/Al_2_O_3_, H_2_O/SiO_2_, and temperature). ^2^ Determined by ICP-OES. ^3^ Determined by XRF.

**Table 2:** Comparison of maximum adsorption capacities of various adsorbents for Pb^2+^ removal.

Material	Temperature (°C)	*Q*_m_ (mg·g^−1^)	Ref.
Na-CHA-p	25	529.1	**This work**
Na-CHA-p	60	546.5	**This work**
Na-CHA-p	80	555.6	**This work**
NaY	25	431.6	[49]
zeolite A		556	[50]
**FAU** zeolite	25	109.9	[43]
zeolite P	25	497.0	[51]
APTES-functionalized zeolite W	35	399.8	[52]
**BEA** zeolite/Fe_3_O_4_ composite	26	139.9	[53]
Mordenite	25	151.3	[54]
MnO_x_-clinoptilolite	23	219.0	[55]
Linde F (K) zeolite/KAlSiO_4_·1.5H_2_O	25	476.1	[56]
CoFe_2_O_4_@CMC@HZSM-5	25	142.8	[57]
SUZ-4 zeolite	25	174.1	[58]

**Table 3:** Comparison of maximum adsorption capacities of various adsorbents for Cd^2+^ removal.

Material	Temperature (°C)	*Q*_m_ (mg·g^−1^)	Ref.
Na-CHA-p	25	188.3	**This work**
Na-CHA-p	60	202.8	**This work**
Na-CHA-p	80	211.0	**This work**
**FAU** zeolite	25	74.1	[43]
zeolite P	24	117.3	[59]
synthetic clinoptilolite	25	44.6	[60]
modified **MOR** zeolite	25	89.7	[61]
modified NaY zeolite	25	23.0	[62]
Na-X	25	238	[63]
natural chabazite	25	120	[64]
zeolite A	24	223.5	[59]
APTES-functionalized zeolite W	35	204.4	[52]
cross-linked chitosan-zeolite	25	102.2	[65]
S-heulandite	25	90.1	[41]

## Data Availability

The original contributions presented in this study are included in the article/Supplementary Material. Further inquiries can be directed to the corresponding author.

## Supplementary Materials

The following supporting information can be downloaded at: https://www.mdpi.com/article/10.3390/molecules31091377/s1. Refs. [39,66] are cited in the Supplementary Materials.
