# Supplementary material for: Green Synthesis of CHA Zeolite from Expanded Perlite Waste for Rapid and Selective Pb2+ and Cd2+ Removal
Source: Molecules. 2026 Apr 22;31(9):1377. doi: 10.3390/molecules31091377 (PMC13165264; doi:10.3390/molecules31091377)
Supplement: Supplementary file 1 [file molecules-31-01377-s001.zip › molecules-4258929-supplementary.pdf]

Supporting Information

Green Synthesis of CHA Zeolite from Expanded Perlite Waste for Rapid and Selective Pb^{2+} and Cd^{2+} Removal

Changchang Fan [1], Binyu Wang [2,3,4], Pan Xu [1], Jiaojiao Lv [1], Haoyang Zhang [1], Zixuan Liang [1] and Wenfu Yan [1,*]

[1] State Key Laboratory of Inorganic Synthesis and Preparative Chemistry, College of Chemistry, Jilin University, Changchun 130012, China; fancc24@mails.jlu.edu.cn (C.F.); xupan24@mails.jlu.edu.cn (P.X.); lvjj20@mails.jlu.edu.cn (J.L.); haoyang22@mails.jlu.edu.cn (H.Z.); liangzx25@mails.jlu.edu.cn (Z.L.)

[2] Changchun Institute of Optics, Fine Mechanics and Physics, Chinese Academy of Sciences, Changchun 130033, China; wangbinyu@ciomp.ac.cn

[3] State Key Laboratory of Advanced Manufacturing for Optical Systems, Changchun 130033, China

[4] University of Chinese Academy of Sciences, Beijing 100049, China

* Correspondence: yanw@jlu.edu.cn

1. Figures and tables

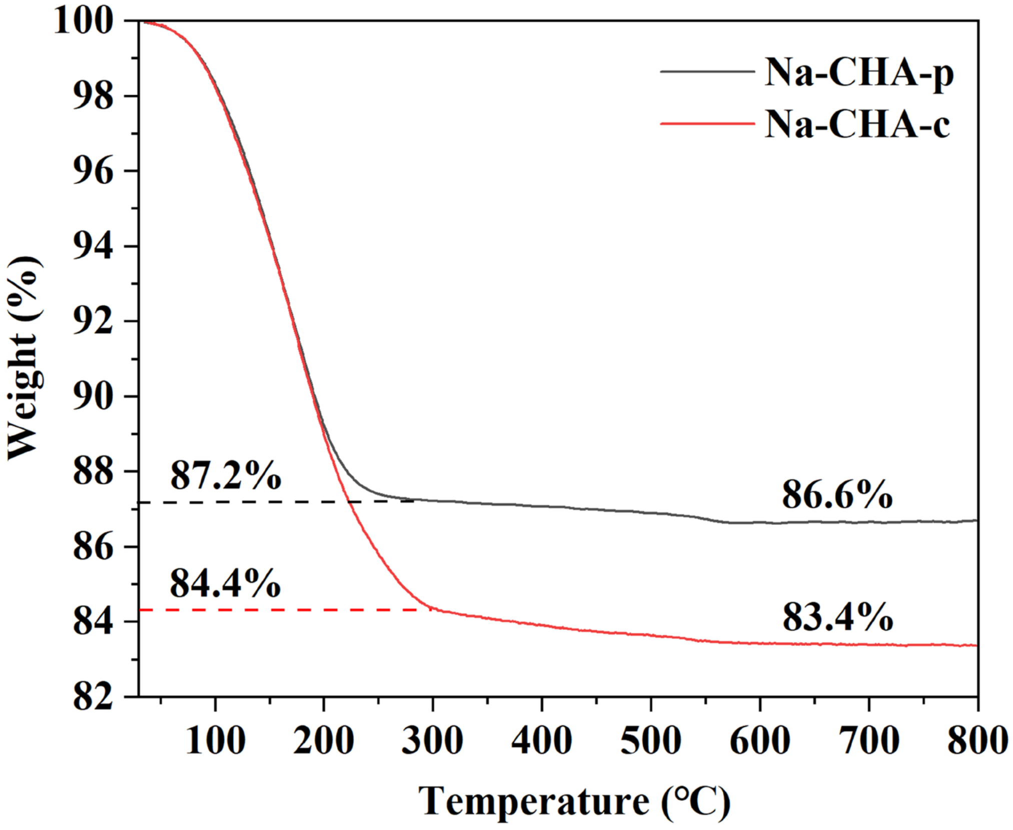

Figure S1. TG-curves of Na-CHA-p and Na-CHA-c.

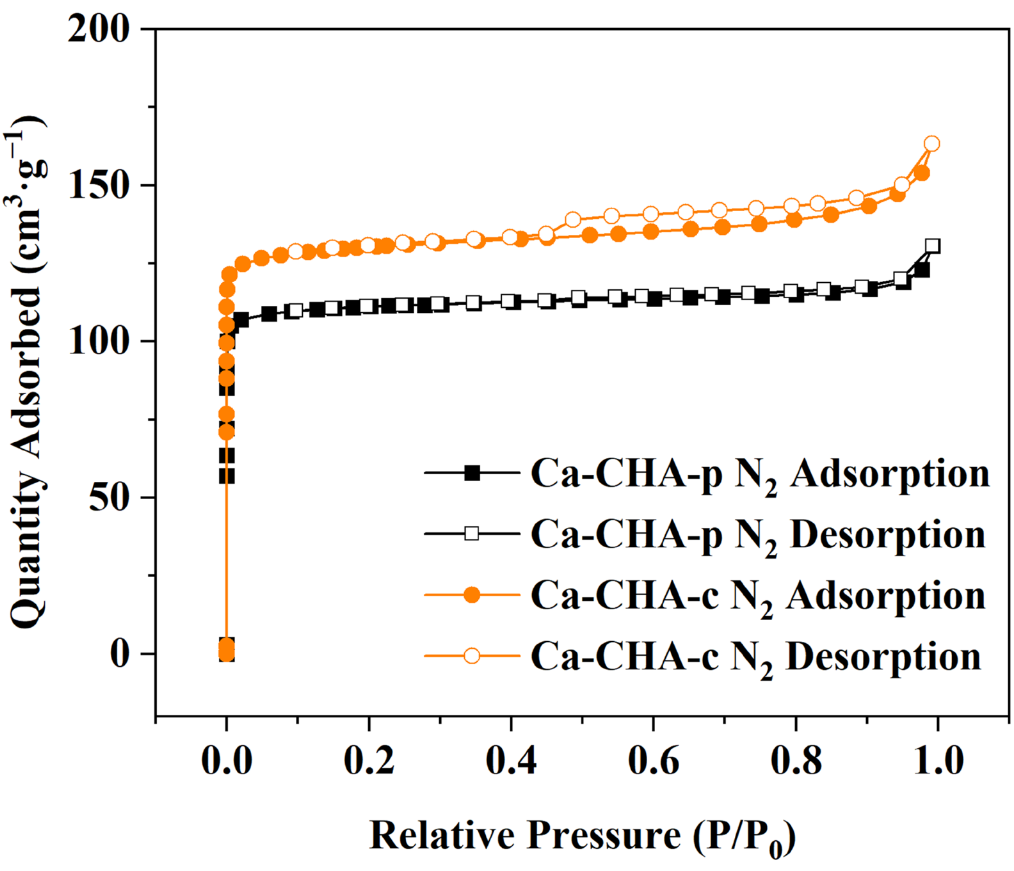

Figure S2. N_2 adsorption–desorption isotherms of Ca-CHA-p and Ca-CHA-c.

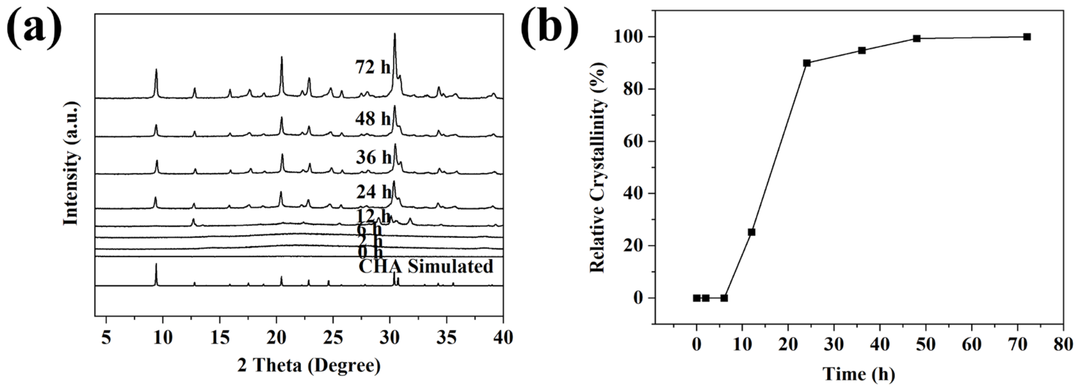

Figure S3. XRD patterns of CHA-p at different crystallization times (a) and relative crystallinity as a function of crystallization time (b).

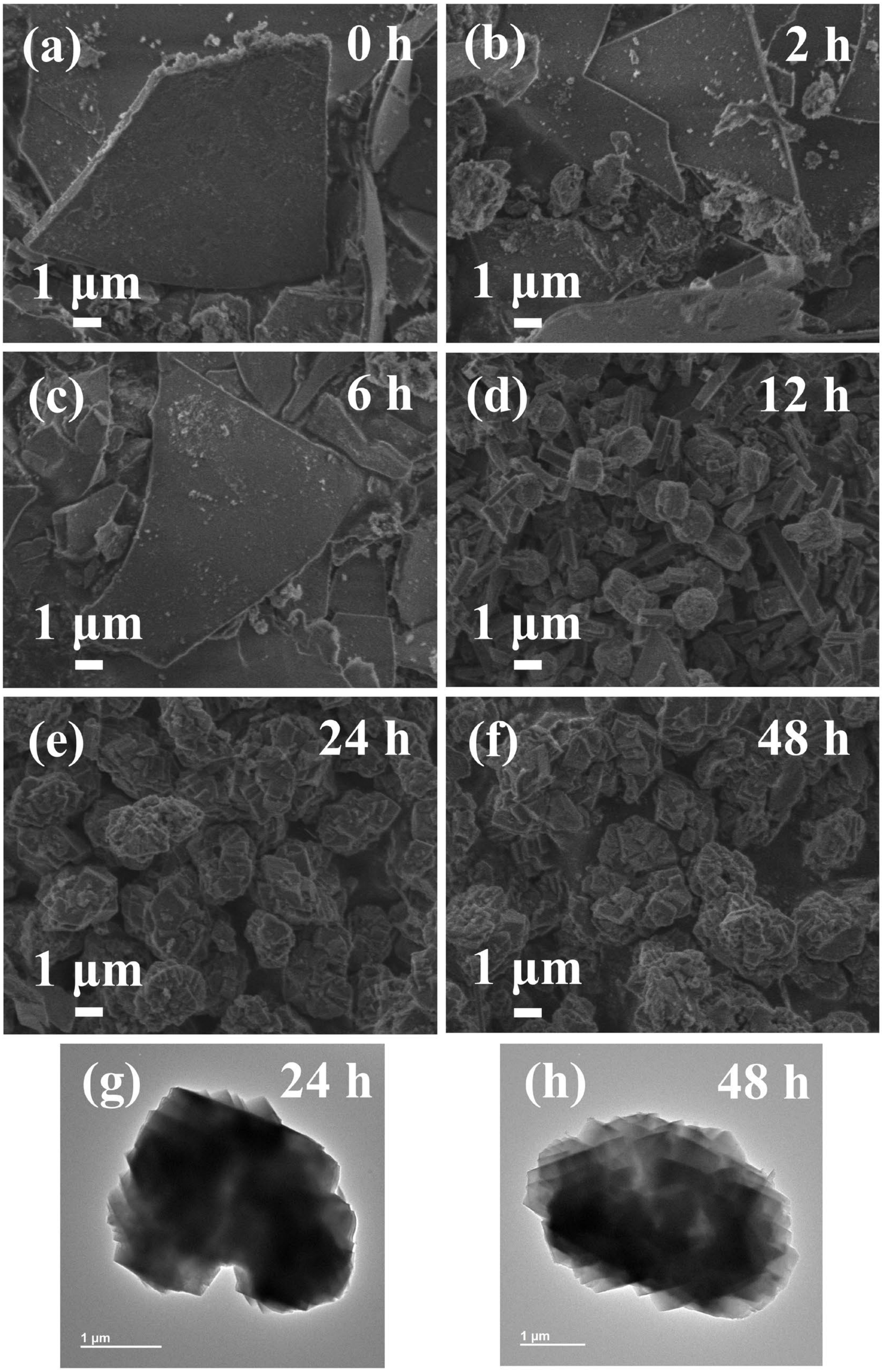

Figure S4. SEM images of CHA-p samples crystallized for 0 h (a), 2 h (b), 6 h (c), 12 h (d), 24 h (e), and 48 h (f); TEM images of CHA-p samples crystallized for 24 h (g) and 48 h (h).

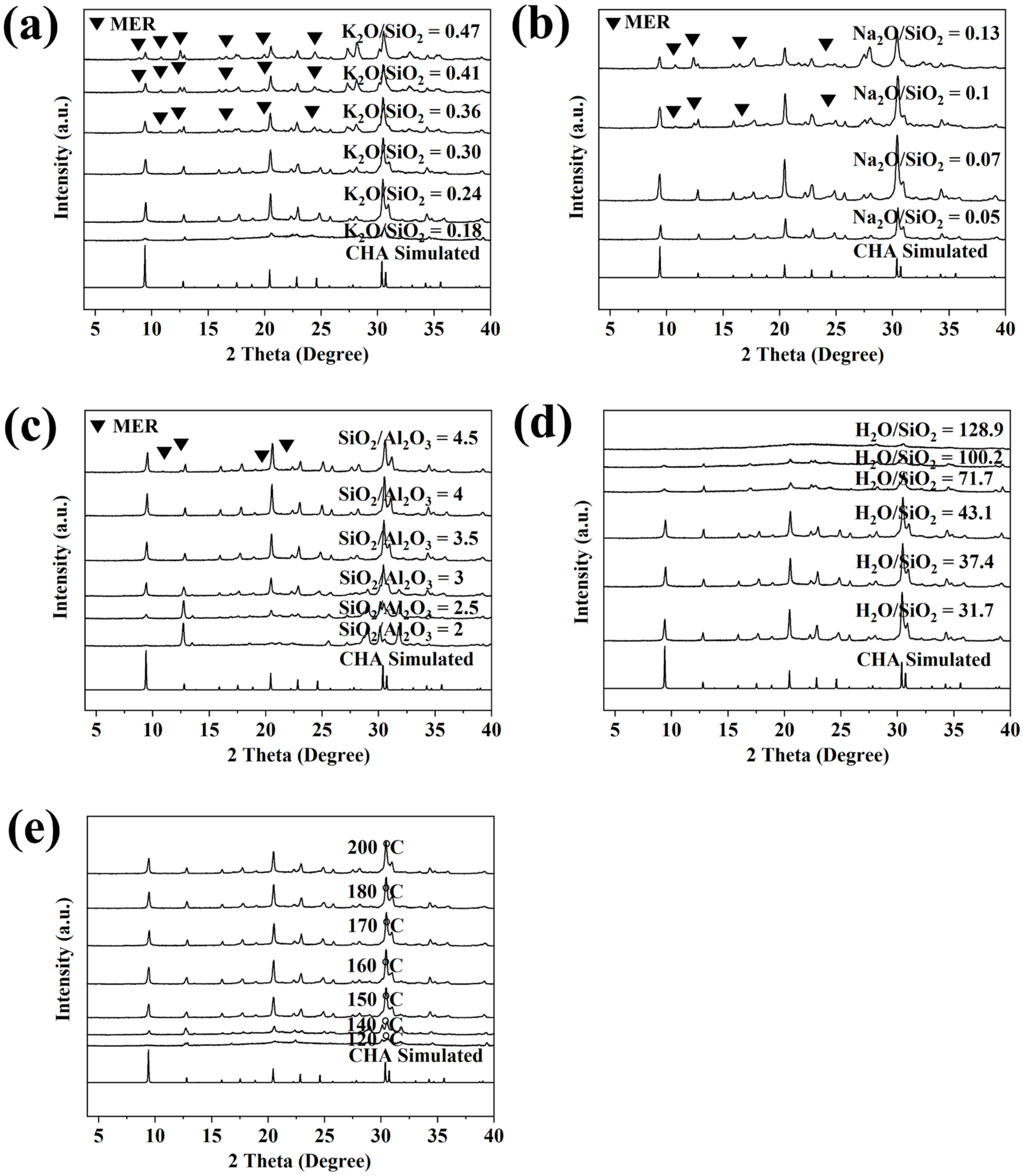

Figure S5. XRD patterns of CHA-p synthesized from precursor gels with various K_2O/SiO_2 ratios (a), Na_2O/SiO_2 ratios (b), SiO_2/Al_2O_3 ratios (c), H_2O/SiO_2 ratios (d), and under different crystallization temperatures (e).

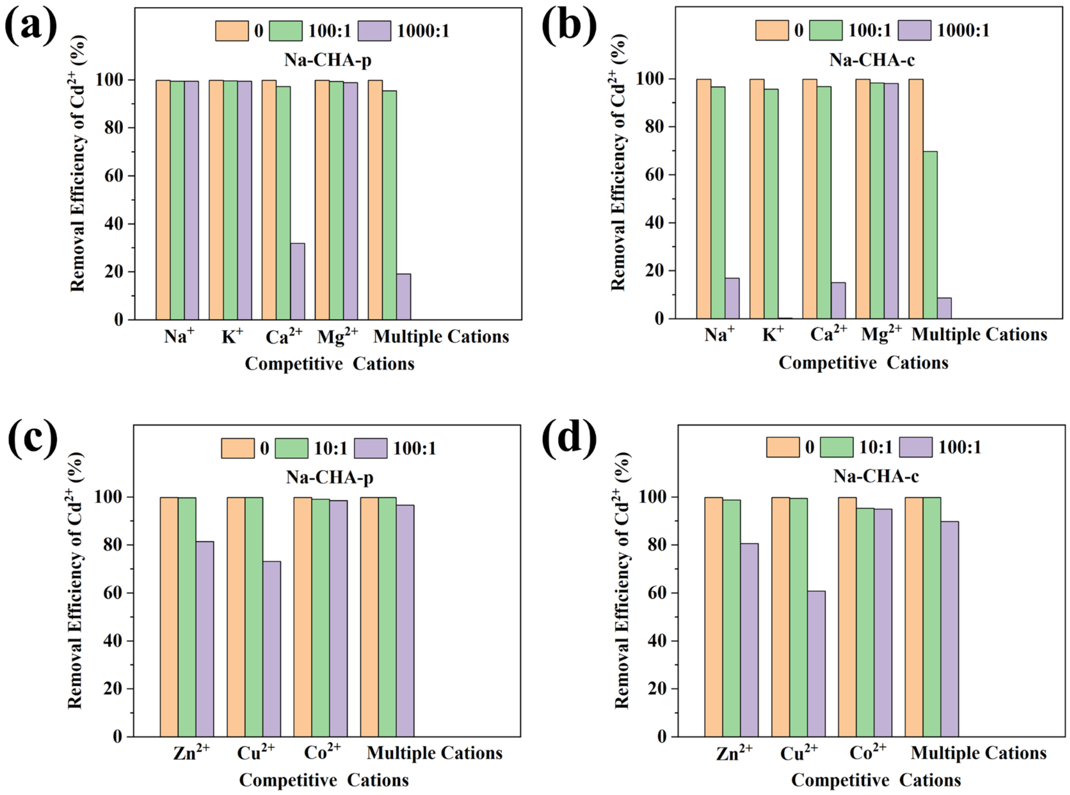

Figure S6. Influence of Na^{+}, K^{+}, Ca^{2+}, and Mg^{2+} cations and their mixture on Cd^{2+} adsorption by Na-CHA-p (a) and Na-CHA-c (b); influence of Zn^{2+}, Cu^{2+}, Co^{2+} cations and their mixture on Cd^{2+} adsorption by Na-CHA-p (c) and Na-CHA-c (d).

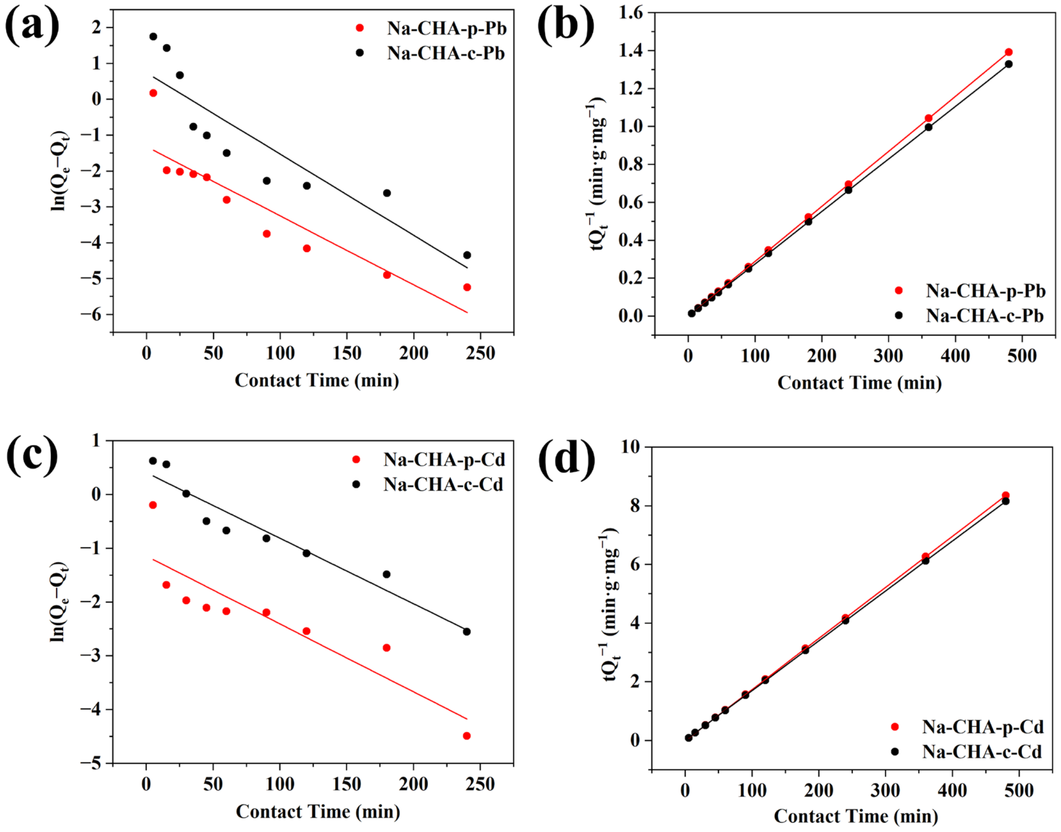

Figure S7. Linear fitting using the pseudo-first-order model for Pb^{2+} (a) and Cd^{2+} (c) and using the pseudo-second-order model for Pb^{2+} (b) and Cd^{2+} (d).

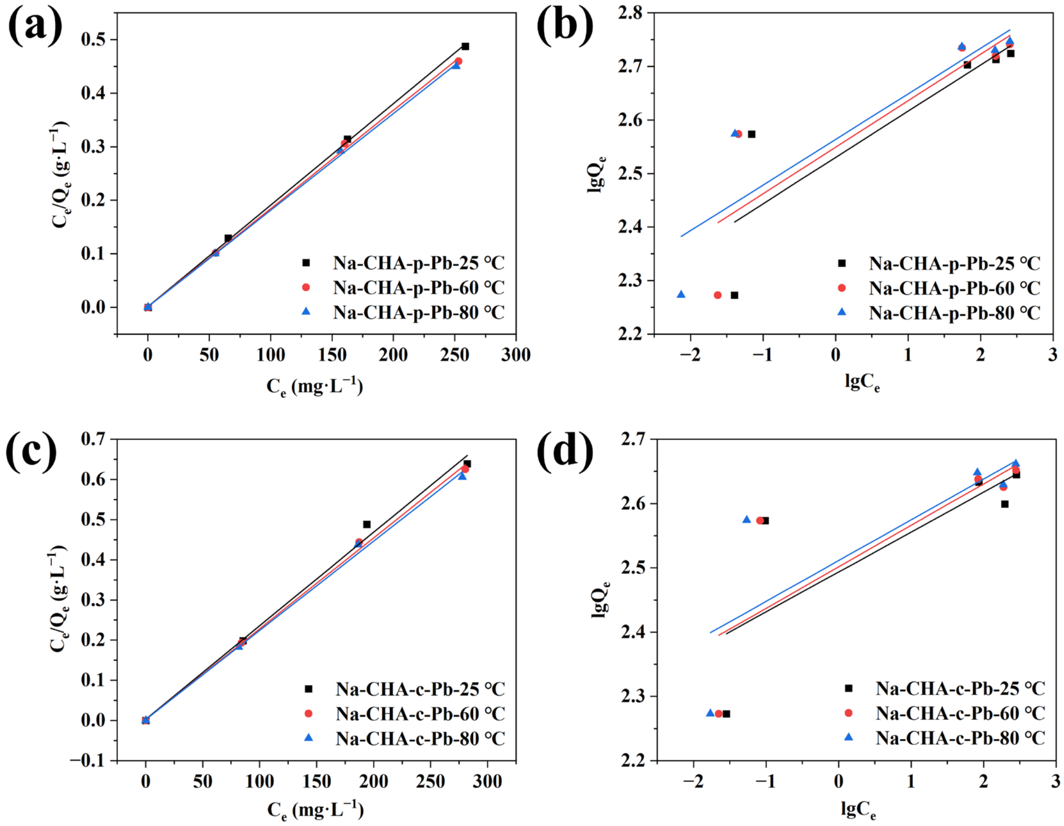

Figure S8. Linear fitting of Pb^{2+} adsorption isotherms on Na-CHA-p (a,b) and Na-CHA-c (c,d) to the Langmuir and Freundlich models, respectively.

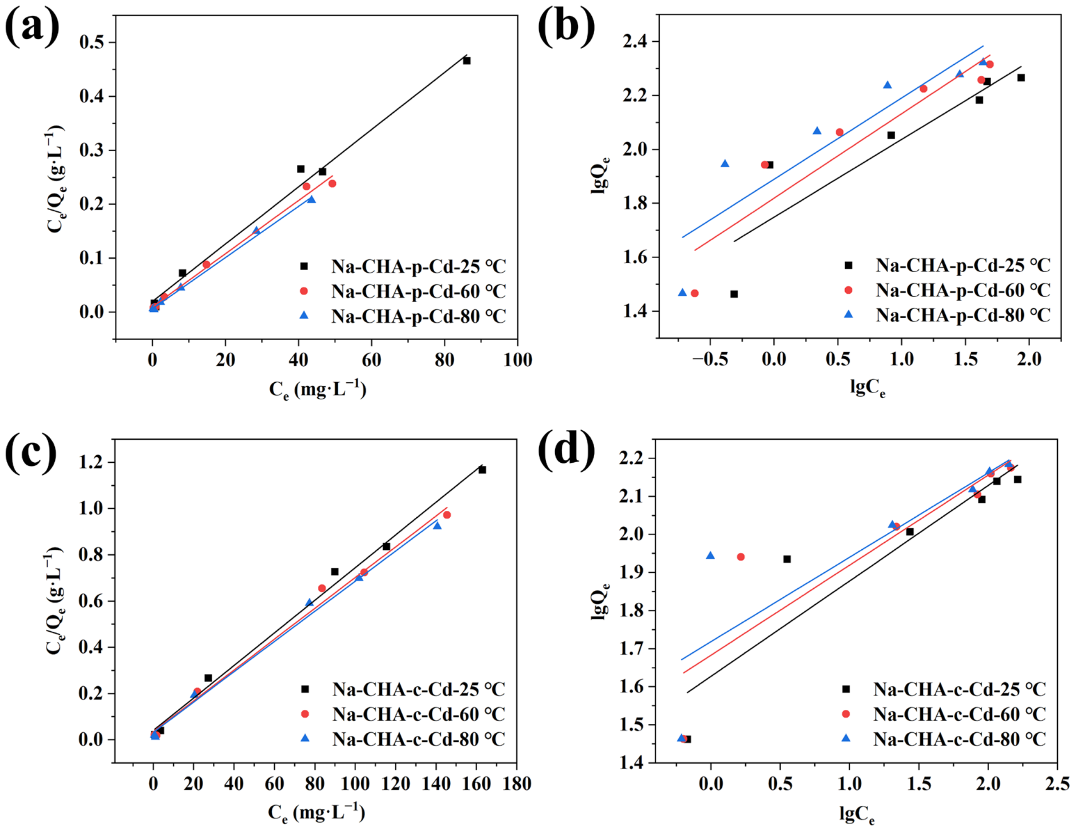

Figure S9. Linear fitting of Cd^{2+} adsorption isotherms on Na-CHA-p (a,b) and Na-CHA-c (c,d) to the Langmuir and Freundlich models, respectively.

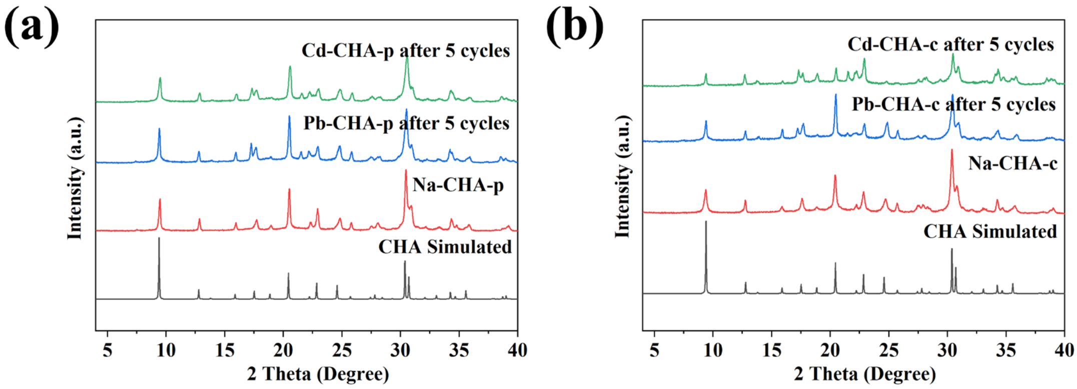

Figure S10. Simulated and experimental XRD patterns of Na-CHA-p (a) and Na-CHA-c (b) after 5 cycles.

Table S1. Elemental composition (wt.%) of expanded perlite waste and Na-CHA-p determined by XRF.

Material	O	Si	Al	Ca	Fe	Ti	K	Na
Expanded perlite waste	49.2	35.5	7.17	0.53	0.49	0.06	4.10	2.62
Na-CHA-p	45.8	25.7	12.8	—	0.29	0.05	0.89	13.2

Table S2. Elemental composition of CHA-p and Na-CHA-p [1].

Sample	Si/Al	K/Al	Na/Al
CHA-p	1.9	0.85	0.11
Na-CHA-p	1.9	0.04	0.92

[1] Determined by ICP analyses.

Table S3. Peak positions and relative areas obtained from the deconvolution of the ^{27}Al MAS NMR spectrum for CHA-p [1].

Species	Peak position (ppm)	Relative area (%)	Assignment
Al^{IV}	58.5	94.7	Framework Al
Al^{VI}	−5.7	5.3	Extra-framework Al

[1] Determined by Lorentzian peak fitting. No Al^{V} species were detected.

Table S4. Textural properties of Ca-CHA-p and Ca-CHA-c.

Sample	Surface area ($m^2 \cdot g^{-1}$) [1]		Pore volume ($cm^3 \cdot g^{-1}$) [2]	
	S_{BET}	S_{micro}	V_{total}	V_{micro}
Ca-CHA-p	456.58	442.76	0.18	0.17
Ca-CHA-c	530.90	508.18	0.22	0.19

[1] S_{BET} was calculated via the multipoint BET method in the P/P_0 range of 0.0000–0.0198 (Ca-CHA-p) and 0.0000–0.0230 (Ca-CHA-c).

[2] V_{total} was determined from the adsorption amount at $P/P_0 \leq 0.90$ according to methodological guidelines [1].

Table S5. Kinetic parameters of Pb^{2+} and Cd^{2+} adsorption on Na-CHA-p and Na-CHA-c.

Groups	Pseudo-first-order model			Pseudo-second-order model		
	R^2	Q_e (mg·g^{-1})	K_1 (min^{-1})	R^2	Q_e (mg·g^{-1})	K_2 (g·mg^{-1}·min^{-1})
Na-CHA-p-Pb	0.795	0.27	0.019	0.999	344.8	0.25
Na-CHA-c-Pb	0.809	2.10	0.023	0.999	361.0	0.033
Na-CHA-p-Cd	0.773	0.32	0.013	0.999	57.4	0.15
Na-CHA-c-Cd	0.923	1.50	0.012	0.999	58.9	0.030

Table S6. Langmuir and Freundlich isotherm parameters of Pb^{2+} adsorption on Na-CHA-p and Na-CHA-c.

Groups		Langmuir isotherm			Freundlich isotherm		
	T (°C)	R^2	Q_m (mg·g^{-1})	K_L (L·mg^{-1})	R^2	n	K_F (mg$^{1-1/n}$·L$^{1/n}$·g^{-1})
Na-CHA-p	25	0.999	529.1	0.82	0.637	11.57	339.09
	60	0.999	546.5	1.19	0.665	11.51	354.38
	80	0.999	555.6	1.18	0.757	11.74	366.45
Na-CHA-c	25	0.994	429.2	0.60	0.475	16.10	311.77
	60	0.998	442.5	0.73	0.529	15.50	317.60
	80	0.997	450.5	0.69	0.528	15.77	324.82

Table S7. Langmuir and Freundlich isotherm parameters of Cd^{2+} adsorption on Na-CHA-p and Na-CHA-c.

Groups		Langmuir isotherm			Freundlich isotherm		
	T (°C)	R^2	Q_m (mg·g^{-1})	K_L (L·mg^{-1})	R^2	n	K_F (mg$^{1-1/n}$·L$^{1/n}$·g^{-1})
Na-CHA-p	25	0.989	188.3	0.26	0.770	3.47	56.17
	60	0.990	202.8	0.49	0.846	3.20	66.14
	80	0.996	211.0	0.68	0.780	3.32	77.68
Na-CHA-c	25	0.993	141.8	0.17	0.827	3.99	42.44
	60	0.989	150.2	0.18	0.739	4.23	48.26
	80	0.991	153.1	0.19	0.675	4.51	52.34

Table S8. Theoretical exchange adsorption capacities calculated from XRF data.

Groups	Theoretical Q_m (mg·g^{-1})
Na-CHA-p-Pb	638.4
Na-CHA-p-Cd	346.3

Table S9. Leaching concentrations of heavy metals from spent adsorbents and comparison with regulatory limits.

Sample	Leached concentration (mg·L^{-1})	EPA regulatory limit (mg·L^{-1}) [1]
Na-CHA-p-Pb	0.21	5.0
Na-CHA-c-Pb	0.48	5.0
Na-CHA-p-Cd	0.11	1.0
Na-CHA-c-Cd	0.25	1.0

[1] Regulatory limit for hazardous waste as defined by US EPA (TCLP Method 1311) [2].

2. References

1. De Lange, M.F.; Vlugt, T.J.H.; Gascon, J.; Kapteijn, F. Adsorptive Characterization of Porous Solids: Error Analysis Guides the Way. *Microporous Mesoporous Mater.* **2014**, *200*, 199–215.
2. Lee, S.; An, J.; Kim, Y.J.; Nam, K. Binding Strength-Associated Toxicity Reduction by Birnessite and Hydroxyapatite in Pb and Cd Contaminated Sediments. *J. Hazard. Mater.* **2011**, *186*, 2117–2122.
